# Retrieval-Guided Transfer Learning for Low-Resource Ebola Drug–Target Affinity Prediction

**DOI:** 10.3390/ijms27188147

**Published:** 2026-09-12

**Authors:** Mubarakah Alotaibi, Nada Al Taweraqi

**Affiliations:** Department of Computer Science, Taif University, Taif 21944, Saudi Arabia; n.difallh@tu.edu.sa

**Keywords:** drug–target affinity prediction, retrieval-guided transfer learning, Ebola virus, BindingDB, virtual screening, drug repurposing

## Abstract

Drug–target affinity (DTA) prediction plays an important role in computational drug discovery; however, its application to emerging infectious diseases such as Ebola remains challenging because of the limited availability of experimentally measured affinity data. To address this low-resource setting, we propose a retrieval-guided transfer-learning framework that leverages BindingDB interactions to improve Ebola DTA prediction. The framework uses a two-stage strategy. In Stage I, source interactions are selected using random sampling, compound-similarity retrieval, protein-similarity retrieval, or hybrid compound–protein retrieval at source-data budgets of 50,000 and 300,000 interactions and combined with Ebola training data to learn transferable representations. In Stage II, the pretrained compound and protein encoders are frozen, while the prediction layers are adapted to the Ebola domain. The framework was implemented with DeepDTA and GraphDTA and evaluated across five random seeds using a scaffold-based split, with conventional machine-learning models and single-stage deep learning as baselines. The best overall configuration, GraphDTA with protein-similarity-guided retrieval at the 50,000-interaction budget, achieved RMSE =0.5498±0.1073, $R^{2}=0.8809\pm 0.0452$, and Pearson =0.9395±0.0237, outperforming the strongest conventional machine-learning model (Extra Trees, RMSE =0.6065±0.0642) and single-stage GraphDTA (RMSE =0.6533±0.0752). Across both source-data budgets and both DTA backbones, all targeted retrieval configurations achieved lower mean RMSE than their corresponding random-retrieval configurations. At the 50,000-interaction budget, matched seed-wise analysis further showed consistent improvements for protein-guided retrieval across all five seeds for both backbones. Retrieval characterization showed stronger target-domain similarity and substantially lower cross-strategy overlap at 50,000 than at 300,000 interactions, while post hoc sequence analysis independently confirmed enrichment of sequence-level relatedness with protein-guided retrieval. Increasing the source-data budget from 50,000 to 300,000 did not uniformly improve predictive performance, indicating that source-data relevance and retrieval selectivity should be considered jointly with source-data quantity. Finally, virtual-screening and approved-drug repurposing case studies across six Ebola virus targets demonstrate the use of the framework for computational prioritization of compound–target hypotheses. Overall, the findings support relevance-guided source-data selection as an effective strategy for transfer learning in low-resource DTA prediction.

## 1. Introduction

### 1.1. Background and Motivation

Ebola virus disease continues to motivate the development of additional antiviral strategies. Although antibody-based therapies have improved treatment, small-molecule therapeutics remain attractive because they can potentially interfere with multiple stages of the viral life cycle [1,2]. Computational methods can reduce the experimental search space by prioritizing compounds before costly laboratory evaluation, and machine learning, virtual screening, and drug repurposing have consequently become important components of Ebola drug-discovery research [3,4].

A complementary problem is quantitative drug–target affinity (DTA) prediction, which estimates continuous binding strength rather than only classifying a compound as active or inactive. Accurate affinity estimates are useful for ranking compound–protein pairs in downstream screening and repurposing applications. Deep DTA models such as DeepDTA and GraphDTA learn compound and protein representations directly from binding data [5,6]; however, their representation-learning capacity is most useful when sufficient experimentally measured interactions are available.

This requirement is restrictive for Ebola because disease-specific affinity data are limited relative to large general-purpose binding databases. Transfer learning provides a natural way to exploit external experimental data, for example, from BindingDB, before adapting a model to the smaller Ebola target domain [7]. The key question addressed in this study is therefore not simply whether external source data help, but which source interactions should be transferred.Random sampling implicitly treats source interactions as equally useful, although their chemical and biological relevance to the target domain can differ substantially.

Motivated by retrieval-guided learning and, in particular, the Stage-Wise Retrieval-Augmented Fine-Tuning (SWAT) principle that task-relevant source samples can improve low-resource adaptation [8], we investigate target-aware source selection for DTA regression. BindingDB interactions are retrieved according to compound similarity, protein similarity, or their combination and compared with random source sampling at two source-data budgets. The resulting two-stage transfer framework is evaluated with DeepDTA and GraphDTA, conventional machine-learning and single-stage deep-learning baselines, retrieval-characterization analyses, matched-seed statistical tests, and two downstream Ebola screening case studies. The relevant literature and the specific gap addressed by this formulation are reviewed in Section 1.2.

### 1.2. Related Work

This subsection reviews the research areas that motivate the proposed framework. We first summarize computational approaches for Ebola drug discovery, followed by representative deep learning models for drug–target affinity prediction and transfer learning in low-resource settings. We then discuss retrieval-guided learning, with particular attention to the SWAT framework that motivated the present approach, and finally identify the research gap addressed in this study.

#### 1.2.1. Computational Drug Discovery for Ebola Virus

Computational drug discovery has been increasingly explored for Ebola virus research, particularly for virtual screening, drug repurposing, and machine learning-based compound prioritization. Kwofie et al. [3] reviewed the roles of artificial intelligence, machine learning, and big data in Ebola virus drug discovery, highlighting the potential of computational models for identifying anti-Ebola compounds while also emphasizing limitations caused by scarce disease-specific data. EBOLApred was developed as a machine learning-based application for predicting small molecules with possible anti-Ebola activity against GP and VP40 using classical classifiers such as random forest, support vector machines, naive Bayes, k-nearest neighbors, and logistic regression [9].

Other studies have focused on drug repurposing and target-specific screening. Lane et al. [10] investigated repurposed candidates such as quinacrine, tilorone, and pyronaridine for Ebola virus disease. Hayat et al. [11] identified potential inhibitors of Ebola VP35 and VP40 from myxobacterial natural products using computational screening. Similarly, Broni et al. [12] used cheminformatics-based screening to identify potential inhibitors targeting the Ebola VP40 protein. Natural-product screening studies have also combined molecular docking, binding-energy analysis, molecular dynamics simulations, and pharmacokinetic evaluation to identify candidate inhibitors against multiple Ebola proteins [13]. Collectively, these studies demonstrate the value of computational screening in Ebola research. However, their primary objective is the identification or prioritization of candidate inhibitors rather than improving the underlying affinity-prediction model with limited Ebola-specific experimental data.

#### 1.2.2. Drug–Target Affinity Prediction

DTA prediction has become an important complementary approach to improving the prioritization of candidate therapeutics identified through computational screening. Rather than simply predicting whether a drug interacts with a target, DTA prediction estimates the continuous binding affinity between a compound and a protein, enabling candidate compounds to be ranked for virtual screening and drug repurposing.

Deep learning has become an important paradigm for DTA prediction because it can automatically learn informative representations of compounds and proteins directly from experimental binding data. Recent studies have explored increasingly sophisticated architectures, including Transformer-based fusion models such as GEFormerDTA [14], enhanced graph neural networks such as GLCN-DTA [15], and pretrained molecular representation frameworks such as PMMR [16], demonstrating continuing advances in DTA prediction.

Despite these advances, DeepDTA [5] and GraphDTA [6] remain representative and widely adopted architectures for DTA prediction. DeepDTA represents compounds as SMILES sequences and proteins as amino acid sequences, learning affinity through convolutional neural networks. GraphDTA extends this approach by representing compounds as molecular graphs processed using graph neural networks while retaining a sequence-based protein representation. Together, these models represent two fundamental paradigms in DTA prediction: sequence-based and graph-based molecular representations.

Since the objective of the present work is to investigate retrieval-guided transfer learning rather than to introduce a new DTA architecture, DeepDTA and GraphDTA were employed as representative backbone models. Their complementary molecular representations enable the proposed retrieval framework to be evaluated across different model families and help isolate the effect of source-data selection from the choice of the underlying prediction architecture.

#### 1.2.3. Transfer Learning for Drug–Target Affinity Prediction

A major limitation of deep DTA models is their dependence on large labeled datasets. This limitation is particularly important for emerging infectious diseases, where experimentally measured compound–protein affinity data are often scarce. Transfer learning addresses this problem by first training models on larger source datasets and subsequently adapting the learned representations to smaller target datasets. In DTA prediction, large public resources such as BindingDB provide an attractive source of experimentally measured protein–ligand binding affinities for learning transferable molecular and protein representations [7,17].

Several recent studies have demonstrated the effectiveness of transfer learning for low-resource drug–target prediction by pretraining models on large source datasets before fine-tuning them on target-specific datasets or protein families [18,19]. However, these approaches typically construct the source dataset from large collections of available interactions without explicitly investigating whether certain source interactions are more beneficial for transfer than others. Consequently, source-interaction selection is generally determined by data availability rather than by the biological or chemical relevance of individual source interactions to the downstream task.

This raises an important question for low-resource DTA prediction: should a model be pretrained on randomly sampled source interactions, or can the retrieval of biologically relevant interactions improve knowledge transfer? The present work investigates this question directly in the context of Ebola drug–target affinity prediction.

#### 1.2.4. Retrieval-Guided Learning and SWAT

Retrieval-guided learning has recently emerged as a strategy for improving transfer learning by selecting source samples that are more relevant to a downstream task. Rather than relying on all available source data or randomly selected samples, retrieval-guided approaches attempt to exploit task-relevant knowledge during model adaptation. In computer vision, retrieval-augmented learning has demonstrated that semantically related source samples can improve adaptation to low-data target tasks [8].

Building on this concept, Liu et al. [8] proposed Stage-Wise Retrieval-Augmented Fine-Tuning (SWAT) for few-shot recognition. Their study demonstrated that simply fine-tuning a model using retrieved samples does not necessarily improve downstream performance because the retrieved data can exhibit two important characteristics: an imbalanced distribution of retrieved samples and a domain gap between the retrieved data and the target few-shot dataset. To address these challenges, SWAT uses a two-stage optimization strategy. The model is first fine-tuned using a mixture of retrieved and target-task samples to learn transferable representations, after which the task-specific classifier is retrained using only the target-task data. This stage-wise procedure is designed to mitigate both the domain gap and the imbalance associated with the retrieved data.

The present work is motivated by the central hypothesis underlying SWAT: not all source samples contribute equally to transfer learning. However, our problem setting differs substantially from few-shot image classification. SWAT retrieves semantically related images from large-scale visual datasets for a classification task, whereas the retrieval unit in the present work is an experimentally measured drug–target interaction and the downstream task is continuous affinity regression. Furthermore, whereas visual semantic similarity provides the principal retrieval signal in SWAT, DTA prediction provides distinct chemical and biological sources of relevance through compound structure and protein sequence.

#### 1.2.5. Research Gap and Difference from Existing Work

Although computational methods have become an important component of Ebola drug discovery, existing studies focus primarily on identifying candidate inhibitors using machine learning classification, molecular docking, natural-product screening, or drug repurposing. These approaches generally prioritize compounds rather than improving the underlying DTA prediction model given the limited availability of experimentally measured Ebola affinity data.

Similarly, existing deep learning DTA models, including DeepDTA and GraphDTA, have demonstrated strong predictive performance on general drug–target affinity benchmarks. However, when transfer learning is employed, models typically rely on large source datasets without explicitly investigating whether the selected source interactions are the most informative for the downstream prediction task. Consequently, the effect of source-data selection on transfer learning remains comparatively underexplored in DTA prediction.

More recently, retrieval-guided learning methods such as SWAT have shown that selecting task-relevant source samples can improve transfer learning in few-shot image recognition. Nevertheless, this concept has not been systematically investigated for drug–target affinity prediction, where the retrieval unit is an experimentally measured drug–target interaction rather than an image, the prediction task is continuous affinity regression rather than classification, and biological and chemical similarity provide natural retrieval criteria.

These limitations identify a specific gap between existing DTA transfer learning and retrieval-guided learning. Although large drug–target databases provide extensive source data for pretraining, it remains unclear whether increasing the amount of source data alone is sufficient or whether selecting a smaller but more target-relevant subset can provide more effective transfer. Moreover, compound and protein similarity represent two distinct forms of relevance in DTA prediction, and their individual and combined effects on transfer performance have not been systematically examined for low-resource Ebola affinity prediction.

### 1.3. Proposed Approach and Contributions

To address the gap identified above, we formulate source-data selection as an explicit component of transfer learning for low-resource Ebola DTA prediction. BindingDB serves as the source domain and experimentally measured Ebola interactions as the target domain. The framework compares random sampling with compound-, protein-, and hybrid-similarity retrieval, evaluates the effect of source-data budget, and tests whether the resulting behavior generalizes across sequence- and graph-based DTA backbones. Methodological details are provided in Section 4, while the corresponding predictive, retrieval-characterization, statistical, and screening results are reported in Section 2.

The main contributions of this work are summarized as follows:

- We propose a retrieval-guided transfer learning framework for low-resource Ebola drug–target affinity prediction using BindingDB as a large-scale source domain.
- We systematically compared conventional random source sampling with compound-similarity, protein-similarity, and hybrid compound–protein retrieval strategies to determine how the relevance of source interactions affects downstream affinity prediction.
- We evaluated the framework using DeepDTA and GraphDTA as representative sequence-based and graph-based DTA architectures and compared the resulting models with conventional machine learning baselines and single-stage deep learning models.
- We investigated the interaction between source-data quantity and retrieval selectivity by evaluating different source-data budgets and characterizing the retrieved subsets in terms of cross-strategy overlap, protein similarity to the Ebola target domain, and compound similarity to the Ebola target domain.
- We evaluated the robustness of retrieval-guided improvements across multiple matched random seeds and statistically assessed the targeted-versus-random retrieval comparisons.
- We demonstrate the practical applicability of the resulting prediction framework through virtual screening of candidate compounds and an approved-drug repurposing case study against representative Ebola virus proteins.

### 1.4. Organization of the Paper

The remainder of this paper is organized as follows. Section 2 presents the experimental results, including comparisons with machine learning and deep learning baselines, the effects of retrieval strategy and source-data size, retrieval-characterization and seed-wise analyses, and the virtual screening and approved-drug repurposing case studies. Section 3 discusses the principal findings, their implications, and the limitations of the proposed approach. Section 4 describes the datasets, retrieval-guided transfer learning framework, source-data selection strategies, model architectures, experimental setup, evaluation metrics, and statistical analysis. Finally, Section 5 presents the conclusions of the study.

## 2. Results

### 2.1. Experimental Context

The proposed retrieval-guided transfer-learning framework was evaluated for Ebola drug–target affinity prediction using BindingDB as the source domain and an Ebola-specific quantitative bioactivity dataset as the low-resource target domain. DeepDTA and GraphDTA were assessed with random, protein-similarity, compound-similarity, and hybrid source-data selection, using 50,000 and 300,000 BindingDB interactions where applicable. The retrieval-guided models were compared with conventional machine-learning baselines and single-stage DeepDTA and GraphDTA models trained only on the Ebola target-domain data. The results are reported across five independent seeds with a scaffold-based split using RMSE, MAE, $R^{2}$, and Pearson correlation; full experimental details are provided in Section 4.

### 2.2. Overall Predictive Performance

Table 1 summarizes the predictive performance of the conventional machine-learning baselines, the single-stage DeepDTA and GraphDTA models, and the retrieval-guided transfer-learning configurations. All results are reported on the held-out test set across five random seeds. Details of the datasets, scaffold-based partitioning, retrieval procedures, training settings, and evaluation protocol are provided in Section 4.

Among the conventional machine-learning baselines, Extra Trees achieved the lowest mean RMSE (0.6065±0.0642), followed closely by Random Forest (0.6125±0.0634). Extra Trees also obtained the lowest MAE (0.4406±0.0284), the highest $R^{2}$ (0.8570±0.0338), and the highest Pearson correlation (0.9272±0.0186) among the conventional models. When trained directly on the Ebola dataset without source-domain transfer, GraphDTA and DeepDTA obtained similar RMSE values of 0.6533±0.0752 and 0.6537±0.0556, respectively. Thus, neither single-stage deep-learning architecture achieved a lower mean RMSE than the strongest conventional machine-learning model.

The performance pattern changed when source-domain transfer was introduced. The best overall configuration was GraphDTA with protein-similarity-guided retrieval at the 50k source-data budget, which achieved an RMSE of 0.5498±0.1073, MAE of 0.3634±0.0654, $R^{2}$ of 0.8809±0.0452, and Pearson correlation of 0.9395±0.0237. In terms of mean RMSE, this corresponds to a reduction of approximately 9.3% relative to Extra Trees and 15.8% relative to single-stage GraphDTA. The lowest MAE among the transfer-learning configurations was obtained by DeepDTA with protein-guided retrieval at 50k (0.3449±0.0511).

At the same 50k source-data budget, protein-guided DeepDTA achieved an RMSE of 0.5576±0.0816, compared with 0.6643±0.0645 for random retrieval, corresponding to a reduction of approximately 16.1%. For GraphDTA, protein-guided retrieval reduced RMSE from 0.6356±0.0741 with random retrieval to 0.5498±0.1073, a reduction of approximately 13.5%. Compound-guided retrieval achieved RMSE values of 0.5957±0.1057 for DeepDTA and 0.5938±0.0786 for GraphDTA, while hybrid retrieval yielded 0.5520±0.0917 and 0.6062±0.0706, respectively.

At the 300k source-data budget, protein-guided GraphDTA again achieved the lowest mean RMSE, with 0.5542±0.0872, followed by compound-guided GraphDTA (0.5769±0.0835) and compound-guided DeepDTA (0.5774±0.0997). Hybrid retrieval produced RMSE values of 0.5885±0.1081 for DeepDTA and 0.5894±0.0624 for GraphDTA, whereas protein-guided DeepDTA achieved 0.6014±0.0989. The random 300k configurations produced higher RMSE values of 0.6168±0.0702 for DeepDTA and 0.6195±0.0954 for GraphDTA.

Comparison across the two source-data budgets showed that increasing the retrieved source set from 50k to 300k did not uniformly reduce prediction error. For GraphDTA with protein-guided retrieval, the mean RMSE was 0.5498 at 50k and 0.5542 at 300k. For DeepDTA, the effect depended on the retrieval strategy: protein-guided retrieval yielded a lower RMSE at 50k (0.5576 versus 0.6014), whereas compound-guided retrieval yielded a lower RMSE at 300k (0.5774 versus 0.5957).

Overall, the lowest mean prediction error was obtained with protein-guided GraphDTA at the 50k source-data budget, while the relative performance of the remaining retrieval strategies varied according to the DTA backbone and source-data budget. The seed-wise consistency of the retrieval effects is examined in Section 2.5, and their relationship to retrieval characteristics and source-data size is discussed in Section 3.2 and Section 3.3.

### 2.3. Retrieval Characteristics and Source-Data Size

To examine how the retrieval strategies affected the composition and target relevance of the selected BindingDB subsets, three complementary characteristics were evaluated. Cross-strategy overlap measures the extent to which the interactions selected by one retrieval strategy are also selected by the other strategies. Protein similarity to Ebola measures the similarity between proteins in the retrieved source interactions and proteins in the Ebola target domain, whereas compound similarity to Ebola measures the structural similarity between retrieved compounds and compounds in the Ebola target domain. These measures characterize the retrieved source subsets independently of downstream prediction performance. The results are reported in Table 2.

At the 50k source-data budget, cross-strategy overlap ranged from 0.0453±0.0003 for random retrieval to 0.0485±0.0004 for protein-guided retrieval. Compound-guided retrieval showed a similar overlap of 0.0484±0.0007. At the 300k budget, overlap values increased to 0.3467±0.0004 for random retrieval, 0.3418±0.0006 for protein-guided retrieval, and 0.3414±0.0004 for compound-guided retrieval.

The similarity measurements showed the expected strategy-specific differences. At 50k, protein-guided retrieval produced the highest protein similarity to the Ebola target domain (0.1793±0.0010), compared with 0.0727±0.0003 for random retrieval and 0.0738±0.0004 for compound-guided retrieval. Compound-guided retrieval produced the highest compound similarity (0.3026±0.0046), compared with 0.2257±0.0026 for random retrieval and 0.2347±0.0026 for protein-guided retrieval.

At the 300k source-data budget, protein-guided retrieval again produced the highest protein similarity (0.0971±0.0008), while the corresponding values were 0.0703±0.0003 for random retrieval and 0.0705±0.0003 for compound-guided retrieval. Compound-guided retrieval produced the highest compound similarity (0.2477±0.0031), compared with 0.2254±0.0026 for random retrieval and 0.2260±0.0029 for protein-guided retrieval.

The relationship between these retrieval characteristics and the predictive performance reported in Section 2.2 is discussed in Section 3.2.

### 2.4. Post Hoc Sequence Characterization of Protein-Similarity Retrieval

**Figure 1:**
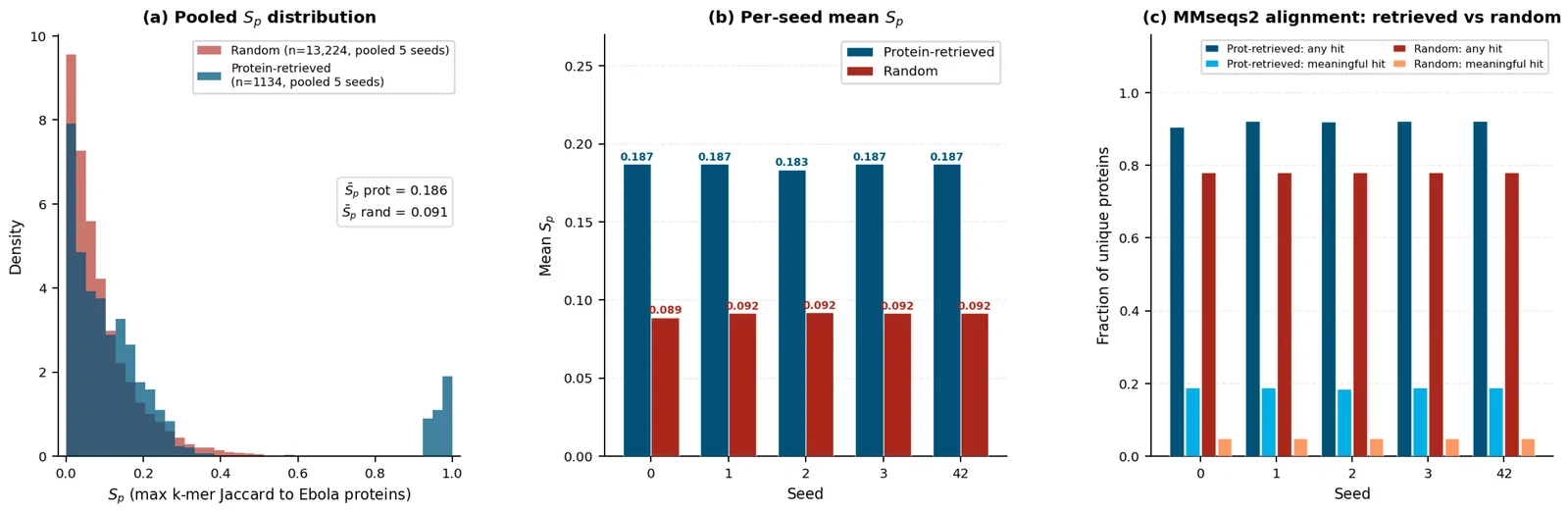
Post hoc sequence characterization of protein-similarity-guided source retrieval at the 50k interaction budget. (**a**) Pooled distribution of maximum protein k-mer Jaccard similarity ($S_{p}$) to proteins in the corresponding Ebola training partition for protein-guided and random retrieval across five seeds. (**b**) Mean $S_{p}$ for each experimental seed. (**c**) Fraction of unique source proteins producing any MMseqs2 alignment hit and a meaningful alignment hit ($E\leq 10^{-3}$ and sequence identity ≥20%) against the corresponding Ebola training proteins. The results are summarized across DeepDTA and GraphDTA experiments.

To further characterize the protein-similarity retrieval strategy, a post hoc sequence analysis was performed using MMseqs2 [20]. The maximum k-mer Jaccard similarity ($S_{p}$) between each retrieved source protein and the Ebola training proteins was examined together with independent sequence-alignment statistics. MMseqs2 easy-search with sensitivity 7.5 was applied to the unique proteins obtained from the protein-similarity and random-retrieval subsets at the 50k source-data budget. Only Ebola proteins from the corresponding training partition were used as alignment targets. The results were summarized across the five seeds (0, 1, 2, 3, and 42) and averaged over the DeepDTA and GraphDTA experiments. The results are shown in Figure 1.

Across seeds, protein-similarity retrieval selected 226.8±2.0 unique source proteins, compared with 2644.8±5.9 unique proteins for random retrieval. The number of unique proteins selected by protein-guided retrieval ranged from 223 to 228 across the five seeds, whereas the corresponding random subsets contained between 2638 and 2651 unique proteins.

As shown in Figure 1a, the pooled $S_{p}$ distributions differed between the two retrieval strategies. The mean maximum k-mer Jaccard similarity was 0.186±0.002 for protein-guided retrieval and 0.091±0.001 for random retrieval. The same pattern was observed for every seed (Figure 1b). The mean $S_{p}$ values for protein-guided retrieval were 0.1871, 0.1870, 0.1835, 0.1870, and 0.1870 for seeds 0, 1, 2, 3, and 42, respectively. The corresponding random-retrieval values were 0.0889, 0.0918, 0.0922, 0.0916, and 0.0918.

The MMseqs2 analysis provided an independent sequence-level characterization of the retrieved proteins (Figure 1c). Across the five seeds, 91.8±0.6% of the proteins selected by protein-guided retrieval produced at least one MMseqs2 alignment hit against an Ebola training protein. Using the predefined meaningful-alignment criterion of $E\leq 10^{-3}$ and sequence identity ≥20%, 18.8±0.1% of the protein-retrieved proteins satisfied this criterion. The corresponding meaningful-hit rate for random retrieval was approximately 5.0%, giving a 3.76-fold difference between the two retrieval strategies.

For the protein-guided subsets, the median sequence identity of the best MMseqs2 hit was 32.5±0.9%, while the median best-hit E-value was 0.685±0.059. Together with the $S_{p}$ distributions and per seed measurements, these alignment statistics provide the sequence-level characteristics of the proteins selected by protein-similarity-guided retrieval. Their relationship to downstream transfer-learning performance is discussed in Section 3.2.

### 2.5. Seed-Wise Consistency of Targeted Retrieval

**Figure 2:**
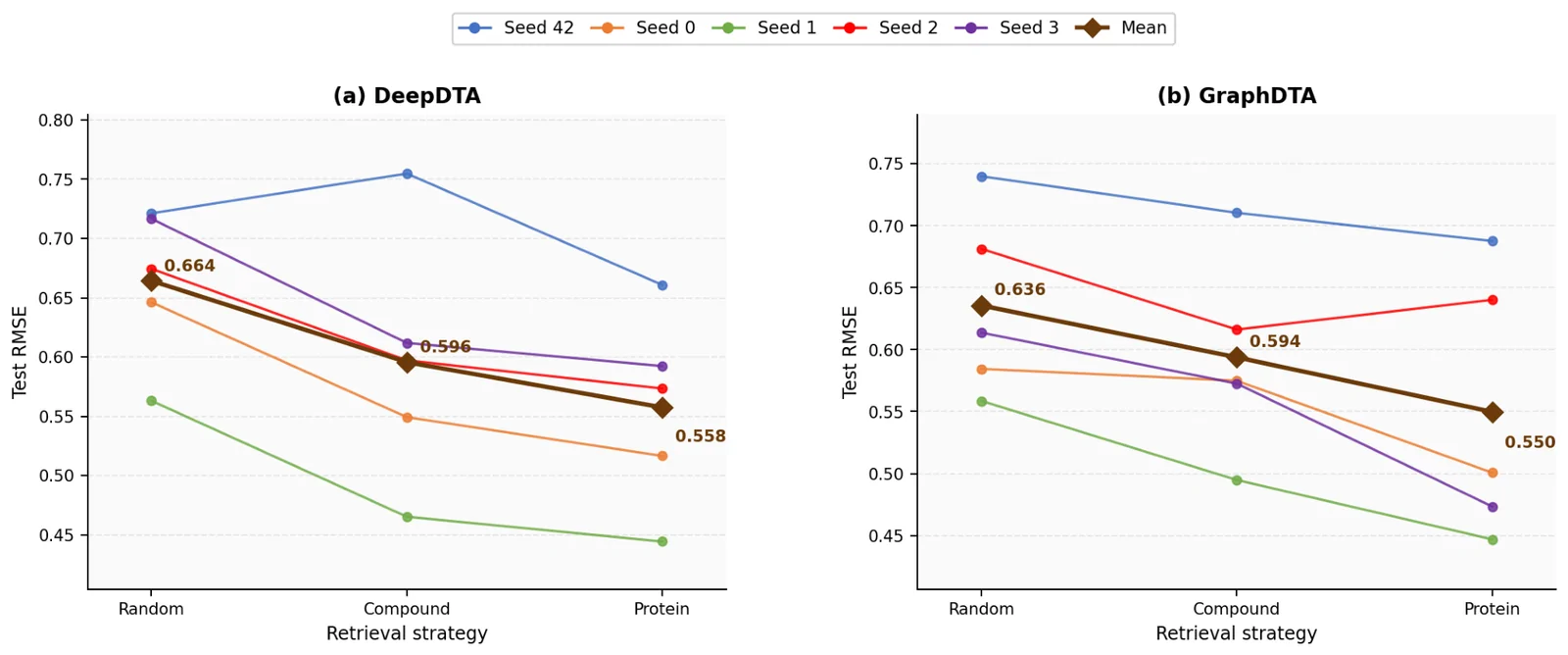
Matched seed-wise RMSE for random, compound-guided, and protein-guided retrieval at the 50k source-data budget for DeepDTA (**a**) and GraphDTA (**b**). Thin trajectories represent individual matched seeds, while the bold trajectory with diamond markers shows the mean across the five runs; mean RMSE values are annotated.

To complement the aggregate performance results reported in Section 2.2, the matched seed-wise results were examined to determine whether the differences between retrieval strategies were consistent across independent runs. The analysis focused on the 50k source-data budget, for which random, protein-guided, and compound-guided retrieval were evaluated with the same source-data size for both DeepDTA and GraphDTA. Figure 2 shows the RMSE values across the five matched seeds together with the mean RMSE for each retrieval strategy.

For DeepDTA, mean RMSE was 0.664 with random retrieval, compared with 0.558 with protein-guided retrieval and 0.596 with compound-guided retrieval, corresponding to reductions of 16.1% and 10.3%, respectively. Protein-guided retrieval achieved lower RMSE than random retrieval in all five matched seeds. Compound-guided retrieval achieved lower RMSE in four of the five seeds; the exception was seed 42, for which the RMSE was 0.755 with compound-guided retrieval and 0.721 with random retrieval.

A similar seed-wise pattern was observed for GraphDTA. Mean RMSE was 0.636 with random retrieval, 0.550 with protein-guided retrieval, and 0.594 with compound-guided retrieval, corresponding to reductions of 13.5% and 6.6% relative to random retrieval. Both protein-guided and compound-guided retrieval achieved lower RMSE than random retrieval in all five matched seeds.

One-sided paired Wilcoxon signed-rank tests were consistent with the seed-wise comparisons. Protein-guided retrieval yielded lower RMSE than random retrieval for both DeepDTA and GraphDTA (p=0.0313 for each). For compound-guided retrieval, the corresponding test yielded p=0.0313 for GraphDTA and p=0.0625 for DeepDTA. The relationship between these seed-wise results, the retrieval characteristics reported in Section 2.3, and source-data size is discussed in Section 3.2.

### 2.6. Case Study I: Virtual Screening of Candidate Compounds

The best-performing transfer-learning configuration, GraphDTA with protein-similarity-guided retrieval using 50,000 BindingDB interactions, was used for the virtual-screening analysis. The final Ebola bioactivity dataset contained 2428 unique compounds. Each compound was evaluated against six principal Ebola protein targets: L protein RNA-dependent RNA polymerase (RdRp), nucleoprotein (NP), envelope glycoprotein (GP), truncated GP, secreted glycoprotein (sGP), and VP35 polymerase cofactor. This resulted in 14,568 unique compound–target predictions (2428×6).

Predictions were generated independently using the five trained models corresponding to seeds 0, 1, 2, 3, and 42. For each compound–target pair, the predicted pAffinity values were summarized using the ensemble mean and standard deviation. Seed agreement indicates the number of seed-specific models in which a compound–target pair appeared among the top-ranked predictions for the corresponding target.

Table 3 presents the 20 highest-ranked compound–target predictions according to ensemble-mean predicted pAffinity. All 20 predictions were associated with sGP. CHEMBL3645734 ranked first with a predicted pAffinity of 9.076±0.300, followed by CHEMBL4858949 (9.070±0.297), CHEMBL4858040 (9.044±0.192), and CHEMBL3645737 (9.029±0.321). Across the Top-20 predictions, ensemble-mean predicted pAffinity ranged from 8.779 to 9.076, while the corresponding standard deviations ranged from 0.121 to 0.525. Seed agreement ranged from 0/5 to 3/5.

Because the global Top-20 predictions were all associated with sGP, the highest-ranked compound was additionally identified separately for each of the six protein targets. Table 4 summarizes these target-specific results together with the number of records associated with each target in the complete target-domain dataset.

The six targets evaluated in the virtual-screening analysis accounted for 2665 of the 2842 compound–protein interactions in the final target-domain dataset. L protein RdRp was represented by 1724 interactions, NP by 779, GP by 55, truncated GP by 45, sGP by 29, and VP35 by 33. The remaining 177 interactions were associated with other less-represented viral protein sequences and were retained in the target-domain modeling dataset but were not treated as separate targets in the downstream virtual-screening analysis.

CHEMBL4114169 was the highest-ranked compound for L protein RdRp, with a predicted pAffinity of 8.543±0.393 and 3/5 seed agreement, and for GP, with 8.245±0.274 and 2/5 agreement. CHEMBL4281745 ranked first for NP (8.376±0.078; 4/5 agreement) and VP35 (8.755±0.491; 3/5 agreement). CHEMBL3645665 ranked first for truncated GP (8.053±0.274; 3/5 agreement), while CHEMBL3645734 ranked first for sGP (9.076±0.300; 2/5 agreement).

The representation of the six screening targets in the target-domain dataset was highly imbalanced, ranging from 29 interactions for sGP to 1724 interactions for L protein RdRp. The concentration of the global Top-20 predictions on sGP was therefore considered together with this variation in target representation. Target-specific rankings, seed agreement, scaffold composition, and the target-normalized sensitivity analysis described in Section 4.7 provide complementary information for interpreting the virtual-screening results.

### 2.7. Case Study II: Approved-Drug Repurposing

The same five-seed GraphDTA ensemble was used to screen 3311 FDA-approved drugs against the six Ebola protein targets, yielding 19,866 drug–target predictions. Ranking consistency and sensitivity to target-specific prediction scales were assessed as described in Section 4.7.

Panel A of Table 5 presents the Top-10 drug–target pairs ranked by ensemble-mean predicted pAffinity. Grazoprevir anhydrous ranked first against sGP, with a predicted pAffinity of 9.478±0.866 and 5/5 seed agreement. Simeprevir ranked second against sGP (9.477±0.584; 5/5 agreement), followed by voxilaprevir against sGP (9.398±0.704; 4/5 agreement). Voxilaprevir against L protein RdRp was the highest-ranked non-sGP prediction, with a predicted pAffinity of 9.118±0.539 and 5/5 seed agreement.

Grazoprevir anhydrous also appeared among the Top-10 predictions against VP35 (9.005±0.968; 5/5 agreement) and L protein RdRp (8.943±0.477; 5/5 agreement). Voxilaprevir ranked ninth against GP (8.784±0.492; 5/5 agreement), while glecaprevir against L protein RdRp completed the raw Top-10 ranking (8.783±0.687; 4/5 agreement).

The predictions were additionally ranked after within-target normalization. As shown in Panel B of Table 5, grazoprevir anhydrous against NP achieved the highest normalized score (z=9.304), with a predicted pAffinity of 8.305 and 5/5 seed agreement. Voxilaprevir against NP ranked second (z=8.901, pAffinity = 8.159; 5/5 agreement), followed by simeprevir against NP (z=7.187, pAffinity = 7.541; 2/5 agreement). The remaining Top-10 normalized predictions included grazoprevir anhydrous against VP35, L protein RdRp, and GP; voxilaprevir against L protein RdRp and GP; and glecaprevir and velpatasvir against NP.

#### Evaluation of Previously Reported Anti-Ebola Compounds

Selected compounds previously investigated in the context of Ebola virus were additionally examined in the screening results. Previous experimental and drug-repurposing studies have investigated small-molecule candidates for anti-Ebola activity, including tilorone and other repurposed compounds [10]. Toremifene, clomiphene, sertraline, bepridil, amiodarone, chloroquine, remdesivir, and daclatasvir were also included as reference compounds in the comparison. Tilorone was evaluated separately because it was not present in the FDA-approved drug screening library.

Table 6 summarizes the predicted affinities and screening ranks of these compounds. Daclatasvir had the highest predicted pAffinity among the reference compounds, with 8.179±1.141 against sGP. Remdesivir and amiodarone also obtained their highest predictions against sGP, with predicted pAffinity values of 6.682±0.735 and 6.175±0.568, respectively.

Among the compounds whose highest predicted values occurred against GP, clomiphene had a predicted pAffinity of 6.100±0.262, followed by tilorone (5.903±0.410), toremifene (5.872±0.411), chloroquine (5.812±0.389), bepridil (5.712±0.304), and sertraline (5.529±0.354).

## 3. Discussion

### 3.1. Retrieval-Guided Transfer in a Low-Resource DTA Setting

The results in Section 2.2 show that the benefit of deep DTA models in the present low-resource setting depends not only on the availability of additional source-domain data but also on how those data are selected. When trained directly on the Ebola dataset, DeepDTA and GraphDTA achieved RMSE values of 0.6537±0.0556 and 0.6533±0.0752, respectively, and neither surpassed the strongest conventional machine-learning model, Extra Trees (0.6065±0.0642). Thus, the use of a more expressive deep architecture alone did not translate into better generalization with the available target-domain data.

Introducing BindingDB interactions during Stage I substantially changed this pattern. The best overall configuration, GraphDTA with protein-similarity-guided retrieval at the 50k source-data budget, reduced the RMSE to 0.5498±0.1073. DeepDTA also benefited from source-domain pretraining, with its best configuration, hybrid retrieval at 50k, achieving 0.5520±0.0917. These results correspond to reductions of approximately 15.8% and 15.6%, respectively, relative to the corresponding single-stage GraphDTA and DeepDTA models.

More importantly, the results consistently favored relevance-guided source selection over random source selection. At 50k, all protein-, compound-, and hybrid-guided configurations achieved lower mean RMSE than the corresponding random configuration for both DTA backbones. The same overall pattern was observed at 300k: every targeted retrieval configuration again achieved a lower mean RMSE than its architecture-matched random counterpart. For example, protein-guided retrieval reduced GraphDTA RMSE from 0.6356 to 0.5498 at 50k and from 0.6195 to 0.5542 at 300k. For DeepDTA, the corresponding protein-guided values were 0.5576 and 0.6014, compared with random-retrieval RMSE values of 0.6643 and 0.6168, respectively.

The matched seed-wise analysis in Section 2.5 strengthens this observation. At the 50k budget, protein-guided retrieval achieved lower RMSE than random retrieval in all five matched seeds for both DeepDTA and GraphDTA. Compound-guided retrieval showed the same direction in all five GraphDTA runs and in four of the five DeepDTA runs. The paired Wilcoxon analysis was significant for protein-guided retrieval with both backbones (p=0.0313), as well as for compound-guided GraphDTA (p=0.0313); the corresponding DeepDTA compound comparison yielded p=0.0625. Although the small number of matched runs limits statistical resolution, the seed-wise results are consistent with the aggregate finding that targeted source selection generally provides a more useful transfer signal than random sampling.

These findings extend the relevance-guided principle motivating SWAT [8] to continuous drug–target affinity prediction. Whereas the original SWAT framework retrieves task-relevant source examples for few-shot visual recognition, the present framework applies the same general principle to experimentally measured drug–target interactions. The results therefore suggest that, in a low-resource DTA setting, the value of the source domain depends not simply on the number of available interactions but on selecting interactions that are informative for the downstream target domain.

### 3.2. Source-Data Relevance Versus Source-Data Quantity

The comparison between the 50k and 300k source-data budgets indicates that increasing the amount of source data does not necessarily produce a corresponding improvement in transfer performance. Rather, the results suggest a trade-off between the amount of source-domain coverage and the degree to which the retrieved interactions remain concentrated around the target domain.

This distinction is particularly clear for protein-guided GraphDTA, which was the strongest configuration at both source-data budgets. Increasing the retrieved set from 50k to 300k changed the RMSE only from 0.5498±0.1073 to 0.5542±0.0872, with the smaller subset retaining a slightly lower mean prediction error. A similar pattern was observed for protein-guided DeepDTA, for which RMSE increased from 0.5576±0.0816 at 50k to 0.6014±0.0989 at 300k. In contrast, compound-guided DeepDTA improved from 0.5957±0.1057 to 0.5774±0.0997 as the budget increased. Thus, the effect of adding more source interactions was not uniform across retrieval strategies or architectures.

The retrieval characteristics in Section 2.3 provide a useful explanation for this behavior. At the 50k budget, protein-guided retrieval increased mean protein similarity to the Ebola domain from 0.0727±0.0003 under random sampling to 0.1793±0.0010. Compound-guided retrieval similarly increased compound similarity from 0.2257±0.0026 to 0.3026±0.0046. At 300k, the corresponding targeted similarities remained higher than random retrieval, but the enrichment was smaller: protein similarity increased from 0.0703±0.0003 to 0.0971±0.0008, while compound similarity increased from 0.2254±0.0026 to 0.2477±0.0031.

The composition of the retrieved subsets changed at the same time. The overlap among retrieval strategies was only approximately 0.045–0.049 at 50k, whereas it increased to approximately 0.341–0.347 at 300k. Consequently, the smaller targeted subsets were more strongly differentiated from random sampling and from one another, while expansion to 300k produced considerably greater sharing of source interactions. This is consistent with the idea that increasing a similarity-ranked retrieval budget progressively incorporates interactions farther down the ranking, thereby increasing coverage while reducing the concentration of the relevance signal.

The post hoc sequence characterization in Section 2.4 provides independent support for the protein-retrieval criterion. Protein-guided retrieval produced a mean $S_{p}$ of 0.186±0.002, compared with 0.091±0.001 for random retrieval. In the MMseqs2 analysis, 18.8±0.1% of proteins in the protein-guided subsets satisfied the predefined meaningful-alignment criterion, compared with approximately 5.0% for random retrieval, corresponding to a ratio of approximately 3.76. These results confirm that the lightweight k-mer similarity measure did not merely select a different subset; it enriched the source data for proteins with greater sequence-level relatedness to the Ebola targets.

At the same time, the MMseqs2 results show that most retrieved proteins were not close sequence matches under the predefined criterion. The advantage of protein-guided retrieval therefore should not be interpreted as arising simply from the presence of large numbers of near-identical Ebola homologues in BindingDB. Rather, $S_{p}$ appears to provide a useful ranking signal that shifts the source distribution toward more target-related interactions without requiring strict sequence homology. Whether the resulting benefit is associated with conserved motifs, structural similarity, functional relationships, or other shared features cannot be determined from the present experiments alone.

Taken together, these findings indicate that source-data quantity and source-data relevance should be considered jointly. A larger source set provides greater interaction coverage, but the additional examples are not necessarily equally informative for the downstream task. Conversely, a smaller similarity-ranked subset can preserve a stronger relevance signal. The present results therefore do not imply that smaller datasets are generally preferable; rather, they show that increasing source-data volume without considering how relevance changes with retrieval depth may yield limited or architecture-dependent gains.

### 3.3. Architecture-Dependent Effects of Retrieval

Although relevance-guided retrieval generally outperformed random source selection, the relative ranking of the targeted strategies differed between DeepDTA and GraphDTA. This indicates that source relevance is not independent of the architecture used to learn from the retrieved interactions.

GraphDTA showed its strongest and most consistent results with protein-guided retrieval. This strategy produced the lowest GraphDTA RMSE at both 50k (0.5498±0.1073) and 300k (0.5542±0.0872), and the 50k configuration was also the best overall model in the study. For DeepDTA, the pattern was less dominated by a single retrieval criterion. At 50k, hybrid retrieval achieved the lowest RMSE (0.5520±0.0917), closely followed by protein-guided retrieval (0.5576±0.0816), whereas compound-guided retrieval was strongest at 300k (0.5774±0.0997). Thus, the most effective targeted strategy depended on both the backbone and the source-data budget.

One possible contributor to this difference is the way the two models encode compounds. DeepDTA represents compounds as SMILES sequences processed by one-dimensional convolutional layers, whereas GraphDTA represents compounds explicitly as molecular graphs [5,6]. Their protein and interaction representations are also learned jointly with these different compound encoders. Consequently, source interactions selected according to protein-, compound-, or combined similarity may influence the learned representation differently across the two architectures.

The particularly strong response of GraphDTA to protein-guided retrieval is notable because its compound branch already captures explicit molecular graph structure. One possible interpretation is that increasing biological relevance on the protein side provides a complementary source-domain signal for this architecture. By comparison, the more variable ordering of protein, compound, and hybrid retrieval for DeepDTA suggests that its sequence-based representation may benefit from a different balance of chemical and protein-level similarity. These explanations remain hypotheses, however, because the present experiments evaluate predictive performance rather than directly probing the internal representations learned by each model.

Accordingly, the results do not support a single universally optimal retrieval rule for DTA transfer learning. The more general conclusion is that targeted selection is preferable to treating source interactions as equally informative, while the specific form of relevance that is most useful can depend on the representation learned by the downstream model. Retrieval strategy, source-data budget, and backbone architecture should therefore be considered jointly when constructing transfer-learning pipelines for low-resource DTA tasks.

### 3.4. Interpretation of the Virtual-Screening Case Studies

The virtual-screening analyses in Section 2.6 and Section 2.7 illustrate how the proposed transfer-learning framework can be used to prioritize compound–target interactions in the data-limited Ebola setting. The two case studies provide complementary perspectives: Case Study I examined compounds associated with the Ebola bioactivity dataset, whereas Case Study II extended the analysis to an approved-drug library and therefore addressed the potential for drug repurposing.

A notable result from Case Study I (Section 2.6) was the concentration of the highest global predictions on sGP (Table 3). This pattern should be considered in relation to the substantial differences in target representation within the Ebola training data. As shown in Table 4, L protein RdRp and NP were supported by considerably more training interactions than sGP, VP35, GP, and truncated GP. The strong sGP predictions therefore illustrate why cross-target comparison based only on the absolute predicted pAffinity can be misleading when the underlying targets are represented by markedly different amounts of data.

The target-specific analysis in Table 4 provides a complementary view of the candidate screen. In particular, CHEMBL4114169 was independently prioritized for both L protein RdRp and GP, while CHEMBL4281745 was prioritized for NP and VP35. Such recurrence across distinct proteins is potentially more informative than a high score against a single target because it identifies compounds whose learned molecular representation is consistently associated with favorable predictions across more than one protein environment. At the same time, the differences in seed agreement and prediction variability show that not all highly ranked interactions were equally stable across independently trained models. These results support considering ensemble consistency together with the predicted affinity when selecting compounds for subsequent investigation.

Case Study II (Section 2.7) showed a related target-dependent effect in the approved-drug screen. The raw affinity ranking in Panel A of Table 5 was dominated by sGP, whereas the within-target normalized ranking in Panel B shifted several of the highest priorities toward NP. This reordering indicates that the magnitude and distribution of model outputs differ across targets. Accordingly, raw and normalized rankings answer slightly different questions. The raw predicted pAffinity represents the direct output of the affinity model, whereas the normalized score measures how strongly a drug stands out relative to other compounds evaluated against the same protein. For this reason, the normalized ranking is most useful as a complementary sensitivity analysis rather than as a replacement for the original predicted affinity.

Another notable feature of the approved-drug analysis was the recurrence of several drugs across different targets. Grazoprevir anhydrous and voxilaprevir, for example, appeared repeatedly among the highest-ranked predictions in both the raw and target-normalized analyses. This repeated prioritization across different protein targets and ranking schemes provides additional support for these compounds as candidates for further investigation. More generally, the comparison between the two ranking approaches demonstrates the value of considering multiple aspects of model output rather than relying solely on the highest numerical affinity score.

The evaluation of previously investigated anti-Ebola compounds in Section 2.7 provides additional context for these findings. In particular, toremifene and clomiphene have previously been reported to inhibit Ebola virus infection [21], while broader screening of approved drugs and molecular probes has identified additional compounds with anti-Ebola activity [22]. Tilorone has also been investigated experimentally as a potential anti-Ebola compound [10,23]. Several reference compounds appeared at different positions in the predicted affinity distribution, rather than being uniformly assigned to the top of the screening list. This is consistent with the fact that drug–target affinity prediction represents only one component of antiviral activity. A compound may exhibit antiviral effects through viral entry, intracellular trafficking, host-cell pathways, or other mechanisms that are not directly represented by the six Ebola protein targets considered in the present study. Consequently, correspondence between predicted compound–protein affinity and previously reported phenotypic antiviral activity is not expected to be exact. The reference-compound analysis is therefore useful primarily for placing the screening predictions within a broader drug-repurposing context.

Taken together, Section 2.6 and Section 2.7 show that compound prioritization in this setting should not depend on a single ranking criterion. Predicted affinity, within-target rank, agreement across independently trained models, target-specific data availability, and recurrence across multiple proteins provide complementary information. Considering these factors jointly offers a more robust strategy for reducing a large screening space to a smaller set of compounds for follow-up. The resulting predictions remain computational priorities, and biochemical and cell-based experiments are required to determine whether the prioritized compound–target interactions translate into measurable antiviral activity.

### 3.5. Limitations and Future Work

Several limitations should be considered when interpreting these findings. First, although transfer learning increases the information available to the models through source-domain interactions, the target-domain dataset remains relatively small. The scaffold-based split assesses generalization to compounds that are structurally distinct from those used for training, but evaluation on an independent Ebola affinity dataset would provide stronger evidence of external generalizability when sufficiently comparable data become available.

Second, the present study evaluates retrieval-guided transfer using DeepDTA and GraphDTA as representative sequence- and graph-based DTA architectures. This design allows the retrieval principle to be examined across two established representation paradigms, but it does not cover the full range of contemporary DTA models. Future work should investigate whether similar retrieval effects are observed with Transformer-based architectures, pretrained molecular representations, protein language models, and more advanced graph-based models.

Third, the retrieval mechanisms intentionally rely on interpretable compound- and protein-similarity measures. Although this facilitates analysis of chemical and sequence-level relevance, these criteria cannot capture all aspects of biological or functional relatedness between the source and target domains. The post hoc MMseqs2 analysis provides independent evidence that protein-guided retrieval enriches the selected source set for sequence-level relatedness to the Ebola targets; however, sequence similarity alone does not establish functional similarity, binding-site conservation, or structural homology. Learned molecular embeddings, pretrained protein representations, binding-site information, protein structural similarity, or jointly learned drug–target retrieval functions could provide richer relevance measures. Complementary structural and functional analyses would also help determine which biological properties underlie the observed benefit of sequence-guided source selection.

Fourth, only two principal source-data budgets, 50k and 300k, were evaluated. Their contrasting performance demonstrates that source-data quantity and retrieval relevance can interact, but these two points are insufficient to determine an optimal retrieval depth. Evaluating intermediate source-data budgets, a broader range of subset sizes, or adaptive similarity thresholds would allow the relationship between coverage and selectivity to be characterized more systematically.

Fifth, the statistical comparisons were based on five matched random seeds. The seed-wise results provide evidence that the principal retrieval effects are reproducible across the evaluated runs, particularly for protein-guided retrieval, which achieved lower RMSE than random retrieval in all five matched seeds for both DTA backbones. However, five runs provide limited statistical resolution, and not every targeted comparison showed the same degree of consistency. A larger number of repeated runs would permit more precise estimation of performance variability, confidence intervals, and effect sizes.

Finally, the virtual-screening and approved-drug repurposing analyses are intended for computational prioritization rather than confirmation of antiviral activity. Ensemble variability and seed agreement provide useful information about ranking stability, but uncertainty remains in the ordering of highly ranked compounds. Moreover, the concentration of high-ranking predictions on particular targets and the changes observed after within-target normalization indicate that raw predicted pAffinity values should be interpreted with consideration of target-specific data availability and prediction distributions. A systematic applicability- domain analysis relative to the chemical space represented during model training was also not included. Predictions for compounds that are chemically distant from the training domain may therefore involve greater extrapolation and should be interpreted cautiously. Future work should incorporate explicit applicability-domain assessment, broader uncertainty characterization, and complementary structure-based analyses. Ultimately, the prioritized compound–target hypotheses require biochemical or cell-based experimental evaluation before conclusions regarding Ebola antiviral activity can be drawn.

Together, these limitations define several directions for extending the framework, including richer retrieval representations, adaptive source-data selection, additional DTA architectures and retrieval budgets, independent target-domain evaluation, and experimentally evaluated screening candidates. Such studies would help determine the extent to which retrieval-guided transfer generalizes to other low-resource drug–target affinity prediction problems beyond Ebola.

## 4. Materials and Methods

### 4.1. Datasets and Preprocessing

Two datasets were used in this study: BindingDB as the large-scale source domain for transfer learning and an Ebola-specific quantitative bioactivity dataset as the low-resource target domain. The datasets contain quantitative bioactivity measurements reported using different assay endpoints, including IC50, EC50, $K_{i}$, and $K_{d}$. To provide a consistent numerical regression target across the source and target domains, concentration-based measurements were standardized to molar units and transformed to a common logarithmic pAffinity scale, as defined below. The resulting datasets were subsequently used within the two-stage retrieval-guided transfer-learning framework.

#### 4.1.1. BindingDB Source Dataset

BindingDB is a publicly available database containing experimentally measured protein–ligand bioactivities collected from the scientific literature [7,17]. The database covers a broad range of proteins and small molecules and includes several types of quantitative measurements, including IC_50_, EC_50_, $K_{i}$, and $K_{d}$.

In the present study, quantitative records with valid compound SMILES, protein sequences, and activity measurements were retained. To harmonize the different measurement types and concentration units, all activity values were first converted to molar units and subsequently represented on a common logarithmic scale, hereafter denoted as pAffinity:

(1) $$\mathrm{pAffinity}=-\log_{10}\left(\mathrm{Activity}\;[M]\right),$$

where M denotes molar concentration (mol L^−1^) and Activity represents the corresponding quantitative measurement (e.g., IC_50_, EC_50_, $K_{i}$, or $K_{d}$). For example, a measurement reported in nanomolar units can equivalently be transformed as $9-\log_{10}\left(\mathrm{Activity}\;\left[\mathrm{nM}\right]\right)$. This transformation places the different quantitative measurements on a common numerical scale, with larger pAffinity values corresponding to lower measured concentrations.

Following preprocessing, the retained BindingDB interactions constituted the source pool from which the Stage I training subsets were selected. Rather than using the entire source pool for every experiment, subsets were constructed according to the random, protein-similarity, compound-similarity, and hybrid retrieval strategies described in Section 4.2.2. BindingDB therefore served exclusively as the external source domain from which transferable compound and protein representations were learned.

#### 4.1.2. Ebola Target Dataset

The target-domain dataset was constructed from Ebola-related quantitative bioactivity data obtained from ChEMBL [24] and PubChem [25]. ChEMBL records were retrieved programmatically using the ChEMBL WebResource Client API. The corresponding target and assay annotations were subsequently audited to establish the provenance of the collected data, including target identifiers, target types, assay types, confidence scores, and BioAssay Ontology (BAO) terms.

The provenance analysis identified 910 quantitative bioactivity records associated with six Ebola-related ChEMBL targets. These records represent the original ChEMBL bioactivity evidence and are summarized in Table 7.

The ChEMBL records were further characterized according to their assay provenance. The majority originated from functional assays with organism-based (BAO_0000218), cell-based (BAO_0000219), or generic assay (BAO_0000019) formats and a confidence score of 1. Binding-assay records included the single-protein format (BAO_0000357) with a confidence score of 9 and cell-based formats (BAO_0000219) with confidence scores of 8–9.

The 910 records reported above refer to the source-level ChEMBL bioactivity data and therefore should not be interpreted as the number of sequence-level instances in the final modeling dataset. During dataset construction, the retrieved bioactivity records were processed, associated with their corresponding Ebola target sequences, and repeated compound–sequence observations were consolidated to obtain the sequence-level representation required by the DTA models. In addition to the ChEMBL-derived data, 55 Ebola glycoprotein (GP) IC_50_ records obtained from PubChem were incorporated into the target dataset. Following the complete preprocessing and integration procedure, the final target-domain dataset comprised 2842 compound–protein pairs involving 2428 unique compounds and 30 protein sequences.

Quantitative measurements in the final dataset were represented using the same pAffinity transformation defined for the BindingDB source dataset in Equation (1). This provided a common numerical response representation across the source and target domains.

The protein-level composition of the resulting dataset is summarized in Table 8.

The dataset was dominated by L protein (RdRp) and nucleoprotein (NP), which together accounted for 2503 of the 2842 pairs (88.1%). The “Other viral proteins” category comprised 177 pairs (6.2%) distributed across 12 less-represented protein sequences. Compared with the BindingDB source domain, the resulting Ebola dataset represents the substantially smaller target domain considered in this study and was used for target-aware source retrieval, model adaptation, validation, and final evaluation according to the leakage-controlled partitioning procedure described in Section 4.4.1.

### 4.2. Proposed Retrieval-Guided Transfer Learning Framework

#### 4.2.1. Overall Framework

**Figure 3:**
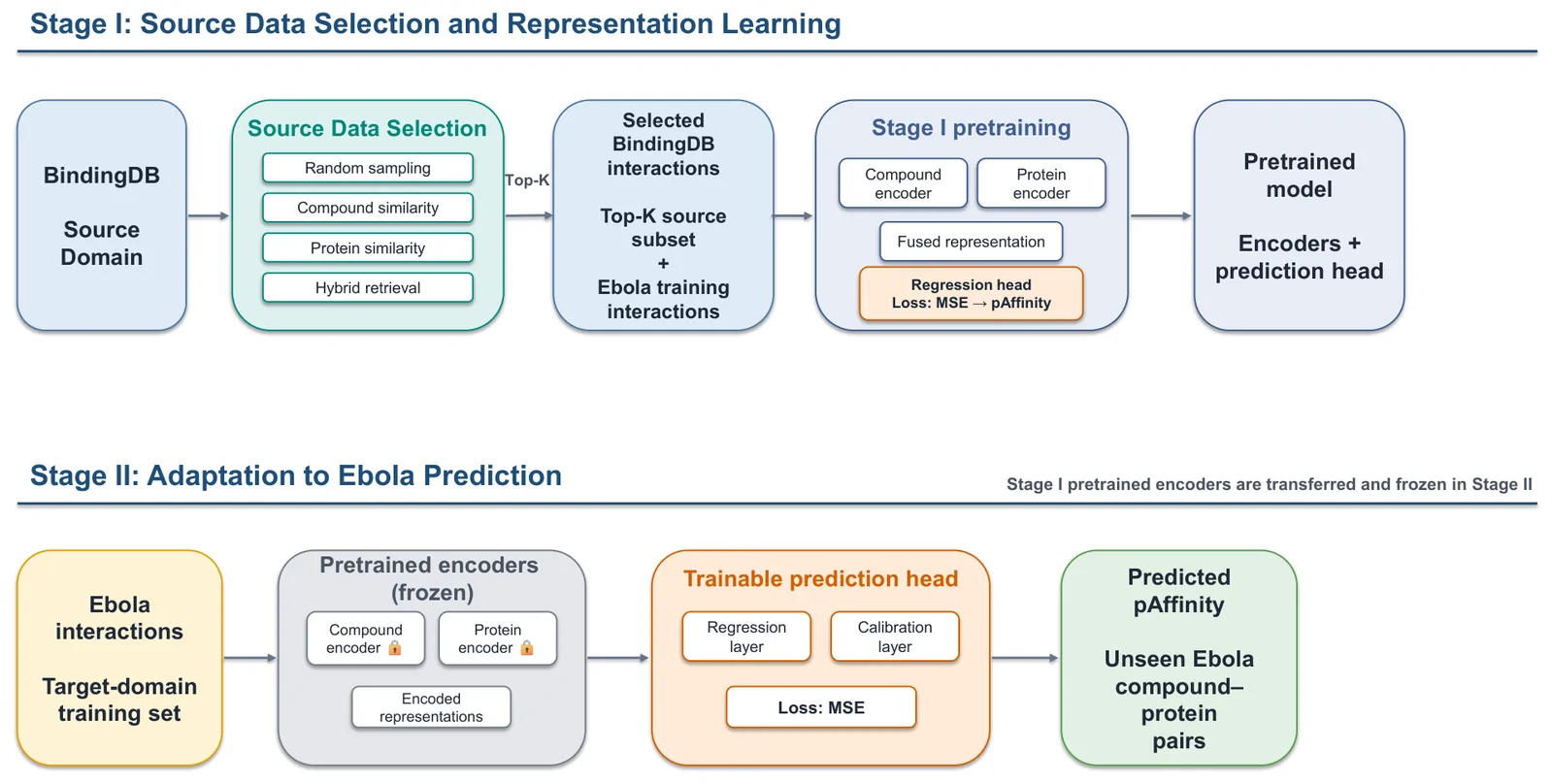
Overview of the proposed two-stage retrieval-guided transfer-learning framework. Stage I selects BindingDB source interactions using random, compound-similarity, protein-similarity, or hybrid retrieval and combines the selected subset with Ebola training interactions for representation learning. Stage II freezes the pretrained compound and protein encoders and optimizes the regression head and calibration layer on the Ebola training domain.

As illustrated in Figure 3, the proposed framework consists of two stages. In Stage I, a BindingDB subset is selected using one of the retrieval strategies defined in Section 4.2.2, combined with the Ebola training partition, and used to learn compound and protein representations with DeepDTA or GraphDTA. In Stage II, the learned representations are transferred to the Ebola prediction task by freezing the pretrained compound and protein encoders while optimizing the regression head and calibration layer on the Ebola training data. The source-selection strategies and the training procedures for the two stages are described in detail in the following subsections.

#### 4.2.2. Stage I: Source-Data Selection

The objective of Stage I source selection is to construct a source training subset containing interactions that can provide useful transferable information for the downstream Ebola prediction task. Let $D_{B}$ denote the BindingDB source pool and $D_{E}^{\mathrm{train}}$ denote the Ebola training partition. A subset of *K* BindingDB interactions is selected according to one of the four retrieval strategies, producing the retrieved dataset $D_{R}$.

The Stage I dataset is then defined as

(2) $$D_{\mathrm{Stage}1}=D_{R}\cup D_{E}^{\mathrm{train}}.$$

Importantly, only the Ebola training partition is used to define the target-domain queries employed for source retrieval. The validation and test partitions are excluded from the retrieval procedure, as described further in Section 4.4.1.

##### Random Sampling

Random sampling serves as the conventional transfer-learning control. A subset of *K* drug–target interactions is selected uniformly at random from BindingDB without considering either chemical similarity between compounds or biological similarity between proteins:

(3) $$D_{R}=\mathrm{RandomSample}(D_{B},K).$$

This strategy provides a reference for determining whether target-aware source selection offers an advantage over simply exposing the model to an equivalent quantity of randomly selected source-domain interactions.

##### Compound-Similarity Retrieval

Compound-similarity retrieval is motivated by the Similarity Property Principle, according to which compounds with similar chemical structures often exhibit similar biological activities and binding behavior [26]. BindingDB interactions containing compounds structurally similar to compounds in the Ebola training partition are therefore preferentially selected.

Each compound is represented using a Morgan fingerprint generated from its SMILES representation. The chemical similarity between a BindingDB compound $c_{i}$ and an Ebola compound $c_{j}$ is measured using the Tanimoto coefficient,

(4) $${\mathrm{Sim}}_{c}(c_{i},c_{j})=\frac{|F_{i}\cap F_{j}|}{|F_{i}\cup F_{j}|},$$

where $F_{i}$ and $F_{j}$ denote the corresponding Morgan fingerprint sets.

For each BindingDB compound, its maximum similarity to the compounds in the Ebola training partition is calculated as

(5) $$S_{c}\left(c_{i}\right)=\max_{e\in E_{c}^{\mathrm{train}}}{\mathrm{Sim}}_{c}\left(c_{i},e\right),$$

where $E_{c}^{\mathrm{train}}$ denotes the set of compounds in the Ebola training partition. BindingDB interactions are ranked in descending order according to $S_{c}$, and the top-*K* interactions are retained:

(6) $$D_{R}=\mathrm{TopK}\left(S_{c},K\right).$$

The resulting source subset is therefore enriched for interactions involving compounds that are structurally related to compounds observed in the target training domain.

##### Protein-Similarity Retrieval

Protein-similarity retrieval is based on the observation that proteins with similar amino acid sequences can share structural and functional characteristics relevant to ligand binding [27,28]. BindingDB interactions involving proteins similar to the Ebola target proteins are therefore prioritized.

Given a protein sequence *P*, a set of overlapping amino-acid *k*-mers is constructed:

(7) $$K\left(P\right)=\left\{\mathrm{all}\;\mathrm{overlapping}\;k\mathrm{-mers}\;\mathrm{extracted}\;\mathrm{from}\;P\right\}.$$

In the experiments, k=3. The similarity between a BindingDB protein $P_{i}$ and an Ebola protein $P_{j}$ is calculated using the Jaccard coefficient,

(8) $${\mathrm{Sim}}_{p}\left(P_{i},P_{j}\right)=\frac{|K\left(P_{i}\right)\cap K\left(P_{j}\right)|}{|K\left(P_{i}\right)\cup K\left(P_{j}\right)|}.$$

For each BindingDB protein, the maximum similarity to the proteins available in the Ebola training partition is then calculated:

(9) $$S_{p}\left(P_{i}\right)=\max_{E\in E_{p}^{\mathrm{train}}}{\mathrm{Sim}}_{p}\left(P_{i},E\right),$$

where $E_{p}^{\mathrm{train}}$ denotes the set of target proteins represented in the Ebola training partition.

BindingDB interactions are ranked according to $S_{p}$, and the top-*K* interactions are selected:

(10) $$D_{R}=\mathrm{TopK}\left(S_{p},K\right).$$

For the retrieval calculation, protein sequences were truncated to a maximum length of 1000 residues. This strategy emphasizes biological relevance at the target-protein level by enriching the source subset for interactions involving proteins with greater sequence similarity to the Ebola targets.

##### Post Hoc Sequence Characterization of Protein-Guided Retrieval

To independently characterize the sequence relationships captured by protein-similarity-guided retrieval, we analyzed the 50,000-interaction protein-guided source subsets together with matched random-retrieval subsets. The analysis was performed independently for each of the five experimental seeds (0, 1, 2, 3, and 42). Within each subset, duplicate protein sequences were removed so that each unique source protein was evaluated once.

To preserve the leakage-control protocol used throughout the study, source proteins were compared exclusively with Ebola proteins from the corresponding training partition. For each unique source protein, the maximum k-mer Jaccard similarity ($S_{p}$) to the Ebola training proteins was recomputed according to Equation (9). This calculation was applied identically to the protein-guided and matched random subsets, enabling direct comparison of the sequence-similarity distributions produced by the two retrieval strategies.

As an independent sequence-based characterization, the unique source proteins were searched against the corresponding Ebola training proteins using MMseqs2 [20]. Searches were performed using the easy-search workflow with a sensitivity setting of 7.5. For each source protein, the best alignment to an Ebola training protein was retained, and its sequence identity and E-value were recorded. In addition to the proportion of source proteins producing any alignment hit, we defined a meaningful alignment as one satisfying both $E\leq 10^{-3}$ and sequence identity ≥20%.

The complete procedure was applied identically to the protein-guided and random-retrieval subsets. For each retrieval strategy, the number of unique source proteins, $S_{p}$ distribution, alignment-hit rate, meaningful-hit rate, median best-hit sequence identity, and median best-hit E-value were calculated for each seed. Summary values are reported as mean ± standard deviation across the five seeds.

##### Hybrid Compound–Protein Retrieval

Compound and protein similarity represent complementary components of a drug–target interaction. The hybrid strategy therefore combines the two retrieval scores to identify BindingDB interactions that jointly reflect chemical and biological relevance.

For each BindingDB interaction *i*, the hybrid score is calculated as

(11) $$S_{h}(i)=\alpha S_{c}(i)+\left(1-\alpha \right)S_{p}(i),$$

where $S_{c}(i)$ and $S_{p}(i)$ denote its compound- and protein-similarity scores, respectively. Equal weighting was used in this study, α=0.5, such that chemical and biological similarity contributed equally to the retrieval score.

The retrieved dataset is then obtained as

(12) $$D_{R}=\mathrm{TopK}\left(S_{h},K\right).$$

This strategy provides a joint source-selection criterion rather than prioritizing either the ligand or target component independently.

#### 4.2.3. Stage I: Representation Learning

The mixed Stage I dataset defined in Section 4.2.2 is used to train either DeepDTA or GraphDTA. DeepDTA learns sequence-based compound and protein representations, whereas GraphDTA combines graph-based compound representations with sequence-based protein representations. In both architectures, the learned drug and protein features are fused and passed to a regression head for continuous affinity prediction.

Stage I training minimizes the mean squared error (MSE) between the predicted and experimentally derived pAffinity values:

(13) $$L_{\mathrm{MSE}}=\frac{1}{N}\sum_{i=1}^{N}{\left({\hat{y}}_{i}-y_{i}\right)}^{2},$$

where $y_{i}$ denotes the experimental bioactivity value standardized to the common pAffinity scale, ${\hat{y}}_{i}$ denotes the corresponding model prediction, and *N* is the number of training samples.

#### 4.2.4. Stage II: Backbone Adaptation

The objective of Stage II is to specialize the Stage I model for Ebola affinity prediction while retaining the representations learned during Stage I. The pretrained compound and protein encoders are therefore frozen, while the regression head and calibration layer remain trainable.

Let θ denote the parameters of the compound and protein encoders and ϕ denote the trainable prediction parameters. During Stage II, the encoder parameters are fixed at their Stage I values:

(14) $$\theta^{*}=\theta_{\mathrm{Stage}1}.$$

The regression head and calibration layer are then optimized using the Ebola training data by minimizing the mean squared error (MSE):

(15) $$\phi^{*}=\arg\min_{\phi }\frac{1}{N}\sum_{i=1}^{N}{\left({\hat{y}}_{i}-y_{i}\right)}^{2},$$

where *N* denotes the number of Ebola training interactions, $y_{i}$ denotes the experimental bioactivity value standardized to the common pAffinity scale for interaction *i*, and ${\hat{y}}_{i}$ denotes the corresponding model prediction.

Freezing the pretrained encoders reduces the number of parameters optimized on the comparatively small target-domain dataset, while allowing the prediction components to adapt to the Ebola-specific pAffinity distribution. The resulting adapted model is subsequently evaluated on the held-out Ebola test partition and used for the downstream compound-prioritization analyses.

### 4.3. DTA Backbone Architectures

Two established deep-learning drug–target affinity (DTA) architectures, DeepDTA [5] and GraphDTA [6], were used as backbone models to evaluate the proposed retrieval-guided transfer learning framework. The principal difference between the two architectures is the representation of the compound: DeepDTA operates on SMILES sequences, whereas GraphDTA represents compounds as molecular graphs. Both models use a sequence-based convolutional encoder for proteins and a fully connected regression head for affinity prediction. The specific configurations used in this study are described below.

#### 4.3.1. DeepDTA

DeepDTA [5] represents both compounds and proteins as sequences. SMILES strings were truncated or padded to a maximum length of 150, while protein sequences were truncated or padded to a maximum length of 1000 amino acids. Both inputs were mapped to 128-dimensional learned embeddings followed by layer normalization.

The compound and protein branches were independently processed by three one-dimensional convolutional layers with 32, 64, and 96 filters, respectively. ReLU activation and batch normalization were applied to the convolutional layers, followed by global max pooling. This produced 96-dimensional representations for both the compound and protein branches.

The two representations were concatenated to form a 192-dimensional joint representation. The two representations were concatenated to form a 192-dimensional joint representation. The regression head consisted of two fully connected hidden layers with 1024 and 512 units, respectively, followed by a single-unit output layer. A dropout rate of 0.1 was applied within the prediction head. Finally, a calibration layer was applied to the regression output to obtain the predicted pAffinity value.

#### 4.3.2. GraphDTA

GraphDTA [6] represents each compound as a molecular graph constructed from its SMILES string. Each atom was described by a 78-dimensional feature vector, and the molecular structure was encoded using three graph-convolutional layers with 64, 64, and 128 output dimensions, respectively, with ReLU activation. Global mean pooling was then applied across the atom representations, followed by a fully connected projection with 128 input and 128 output units, yielding a 128-dimensional compound representation.

The protein branch used amino acid sequences truncated or padded to a maximum length of 1000 residues. Protein tokens were mapped to 128-dimensional learned embeddings and processed by three one-dimensional convolutional layers with 32, 64, and 96 filters, respectively. ReLU activation and global max pooling were then applied, producing a 96-dimensional protein representation.

The compound and protein representations were concatenated to form a 224-dimensional joint representation. As in DeepDTA, the compound and protein representations were concatenated to form a 224-dimensional joint representation. The same regression-head and calibration-layer configuration described for DeepDTA was then applied to obtain the predicted pAffinity value.

### 4.4. Experimental Setup and Evaluation Protocol

All configurations were evaluated using a common experimental protocol. Following data collection and preprocessing, the final Ebola target-domain dataset contained 2842 valid drug–target interactions. Experiments were repeated using five independent seeds (0, 1, 2, 3, and 42) under the scaffold-based evaluation protocol. Performance metrics were calculated independently for each run and subsequently summarized as mean ± standard deviation.

#### 4.4.1. Data Partitioning and Leakage Control

A scaffold-based strategy was used to partition the Ebola dataset into approximately 70% training, 15% validation, and 15% test data. Bemis–Murcko molecular scaffolds were used as grouping variables so that compounds belonging to the same scaffold group were not distributed across different partitions. Explicit checks were additionally performed to ensure that compounds and exact drug–target pairs did not overlap between the training, validation, and test partitions.

Because scaffold groups vary in size, the exact number of interactions in each partition varied slightly among random seeds. The training partition was used for model fitting and source-data retrieval, the validation partition was used for early stopping and model selection, and the test partition was reserved exclusively for final evaluation.

The target-domain split was generated *before* source retrieval. Only compounds and proteins represented in the Ebola training partition were available to the retrieval procedure. Neither the validation nor the test interactions were used to rank or select BindingDB interactions, preventing information from the held-out target partitions from directly influencing source-data selection.

For Stage I checkpoint selection, the retrieved BindingDB interactions and Ebola training interactions were further divided into an internal training and validation split using compound-grouped sampling. Ten percent of this mixed Stage I dataset was used for internal validation, with compounds grouped so that the same compound did not occur in both portions. This internal validation set was used only for Stage I early stopping and was independent of the held-out Ebola validation and test partitions.

#### 4.4.2. Source-Data Budgets

Two source-data budgets were evaluated: 50,000 and 300,000 BindingDB interactions. The same budgets were applied to the random, protein-similarity, and compound-similarity strategies, while hybrid retrieval was evaluated at the 300,000-interaction setting.

The comparison between 50k and 300k was designed to distinguish the effect of source-data quantity from the effect of source-data relevance. Random selection provides a quantity-based control, whereas protein- and compound-guided retrieval preferentially select source interactions related to the Ebola training domain.

#### 4.4.3. Optimization and Training Settings

DeepDTA and GraphDTA were implemented in PyTorch (version 2.12.0) and optimized using AdamW. Mean squared error was used as the training objective. A cosine-annealing learning-rate scheduler was employed, with the minimum learning rate set to one twentieth of the corresponding initial learning rate. The gradient norm was clipped to a maximum value of 1.0.

For Stage I representation learning, the initial learning rate was $1\times 10^{-3}$, and the weight decay was $1\times 10^{-4}$. Training used a batch size of 256 for a maximum of 100 epochs. Early stopping was performed according to validation RMSE with a patience of 20 epochs, and the checkpoint achieving the lowest validation RMSE was retained.

For Stage II Ebola-specific adaptation, AdamW was used with an initial learning rate of $5\times 10^{-4}$, weight decay of $1\times 10^{-4}$, and a batch size of 64. Training was performed for a maximum of 100 epochs, with early stopping based on validation RMSE using a patience of 15 epochs. The checkpoint with the lowest validation RMSE was used for final evaluation.

The optimization configuration was kept fixed between DeepDTA and GraphDTA and across the retrieval strategies to avoid confounding source-selection effects with changes in the training procedure.

For reproducibility, NumPy (version 2.4.6) and PyTorch (version 2.12.0) random-number generators were initialized using seeds 0, 1, 2, 3, and 42. Deterministic cuDNN execution was enabled and cuDNN benchmarking was disabled to reduce nondeterministic variation across repeated runs.

#### 4.4.4. Machine-Learning Baselines

Seven conventional regression algorithms were included as baselines: Random Forest, Extra Trees, Gradient Boosting, support vector regression (SVR), Ridge regression, ElasticNet, and *k*-nearest neighbors (KNN). Unlike the proposed transfer-learning framework, these models were trained exclusively using the Ebola target-domain training data and did not use BindingDB pretraining or retrieval.

To provide a common representation, compounds were encoded using 1024-bit Morgan fingerprints and protein sequences were represented using 20-dimensional amino acid composition (AAC) descriptors. Concatenating the two representations produced a 1044-dimensional feature vector for each drug–target interaction.

Random Forest and Extra Trees each used 500 trees, and a minimum of two samples per leaf. Gradient Boosting used 300 estimators, a learning rate of 0.05, maximum tree depth of 4, and subsampling ratio of 0.8. SVR used an RBF kernel with C=10, ϵ=0.1, and the default scale-dependent kernel coefficient. Ridge regression used α=1.0, whereas ElasticNet used α=0.1, an $\ell_{1}$ ratio of 0.5, and a maximum of 5000 optimization iterations. KNN used seven neighbors with distance-weighted prediction. Standardization was applied before fitting the scale-sensitive SVR, Ridge, and ElasticNet models.

#### 4.4.5. Single-Stage Deep-Learning Baselines

DeepDTA and GraphDTA were additionally evaluated without source-domain transfer. In these experiments, each architecture was initialized and trained directly on the Ebola training partition without access to BindingDB or any retrieval procedure. The same scaffold-based training/validation/test partitions were used.

The single-stage models used the same molecular and protein representations and the same backbone configurations described in Section 4.3. These experiments isolated the contribution of source-domain transfer by allowing the proposed framework to be compared with the corresponding DTA architecture trained solely on the limited target-domain data.

### 4.5. Evaluation Metrics

Predictive performance was evaluated using root mean squared error (RMSE), mean absolute error (MAE), the coefficient of determination ($R^{2}$), and Pearson’s correlation coefficient (*r*). Let $y_{i}$ denote the experimental bioactivity value standardized to the common pAffinity scale, ${\hat{y}}_{i}$ the corresponding predicted pAffinity value, $\bar{y}$ the mean observed affinity, $\bar{\hat{y}}$ the mean predicted affinity, and *n* the number of test interactions, $\bar{y}$ the mean observed affinity, $\bar{\hat{y}}$ the mean predicted affinity, and *n* the number of test interactions.

RMSE was calculated as

(16) $$\mathrm{RMSE}=\sqrt{\frac{1}{n}\sum_{i=1}^{n}{\left(y_{i}-{\hat{y}}_{i}\right)}^{2}}.$$

MAE was calculated as

(17) $$\mathrm{MAE}=\frac{1}{n}\sum_{i=1}^{n}\left|y_{i}-{\hat{y}}_{i}\right|.$$

The coefficient of determination was calculated as

(18) $$R^{2}=1-\frac{\sum_{i=1}^{n}{\left(y_{i}-{\hat{y}}_{i}\right)}^{2}}{\sum_{i=1}^{n}{\left(y_{i}-\bar{y}\right)}^{2}}.$$

Pearson’s correlation coefficient was calculated as

(19) $$r=\frac{\sum_{i=1}^{n}\left(y_{i}-\bar{y}\right)\left({\hat{y}}_{i}-\bar{\hat{y}}\right)}{\sqrt{\sum_{i=1}^{n}{\left(y_{i}-\bar{y}\right)}^{2}}\sqrt{\sum_{i=1}^{n}{\left({\hat{y}}_{i}-\bar{\hat{y}}\right)}^{2}}}.$$

Lower RMSE and MAE indicate smaller prediction errors, whereas larger $R^{2}$ and Pearson’s *r* indicate stronger agreement between predicted and experimentally measured affinities. Each metric was calculated separately for every seed and subsequently reported as mean ± standard deviation over the five runs.

### 4.6. Statistical Analysis

To examine whether the improvements obtained through targeted retrieval were consistent across repeated runs, a paired seed-wise statistical analysis was conducted using the 50k source-data setting. For each architecture, the test RMSE obtained using protein- and compound-guided retrieval was compared separately with the RMSE obtained using random retrieval for the same five random seeds (0, 1, 2, 3, and 42). Each comparison therefore consisted of five matched observations.

Because the number of paired observations was small and normality of the within-seed RMSE differences could not be assumed, the one-sided Wilcoxon signed-rank test was used. For each targeted retrieval strategy, the null and alternative hypotheses were

(20) $$H_{0}:{\mathrm{RMSE}}_{\mathrm{targeted}}\geq {\mathrm{RMSE}}_{\mathrm{random}},$$

and

(21) $$H_{1}:{\mathrm{RMSE}}_{\mathrm{targeted}}<{\mathrm{RMSE}}_{\mathrm{random}}.$$

Four planned comparisons were performed: DeepDTA protein versus random, DeepDTA compound versus random, GraphDTA protein versus random, and GraphDTA compound versus random. Statistical significance was evaluated at α=0.05. The corresponding seed-wise results and *p*-values are reported in Section 2.3. The comparisons used the same matched five seeds for each strategy, rather than treating repeated runs as independent observations.

### 4.7. Screening Robustness and Ranking Analysis

To characterize the robustness of the compound-prioritization results, predictions were examined across independently trained models and, where applicable, using target-specific score normalization. Chemical scaffold composition was additionally characterized to assess structural redundancy among highly ranked compounds.

For the seed-wise consensus analysis, compound–target predictions were ranked independently for each of the five experimental seeds (0, 1, 2, 3, and 42). For each compound–target pair, we recorded the number of seed-specific rankings in which the pair appeared among the Top-10. This consensus count was used as a descriptive measure of ranking consistency across experimental runs, with higher counts indicating that a prediction was repeatedly prioritized across independently trained models.

Chemical diversity among highly ranked candidate compounds was characterized using Bemis–Murcko scaffolds. The scaffold composition of the highest-ranked compounds was examined to identify repeated structural frameworks and to provide context for the chemical diversity of the prioritized candidates. Scaffold information was used as a descriptive characterization of the ranking rather than as a constraint on model prediction.

Because the magnitude of predicted pAffinity may differ systematically among the six Ebola protein targets—L protein RNA-dependent RNA polymerase (RdRp), nucleoprotein (NP), envelope glycoprotein (GP), truncated GP, secreted glycoprotein (sGP), and VP35 polymerase cofactor—a target-normalized ranking was additionally evaluated as a sensitivity analysis. Predicted pAffinity values were standardized independently within each target according to

(22) $$z_{ij}=\frac{{\hat{y}}_{ij}-\mu_{j}}{\sigma_{j}},$$

where ${\hat{y}}_{ij}$ denotes the predicted pAffinity of compound *i* for target *j*, and $\mu_{j}$ and $\sigma_{j}$ denote the mean and standard deviation, respectively, of the predicted pAffinity values for target *j*. Compound–target pairs were ranked using both the raw predicted pAffinity values and the target-normalized scores. The normalized ranking was used only as a sensitivity analysis to evaluate the influence of target-specific prediction scales on cross-target prioritization and was not treated as a replacement for the original pAffinity predictions.

Finally, compounds with previously reported anti-Ebola activity were examined to provide context for the approved-drug prioritization results. These reference compounds included toremifene, clomiphene, sertraline, bepridil, and tilorone. Toremifene, clomiphene, sertraline, and bepridil, including available salt or free-base forms, were present in the approved-drug library. Tilorone was absent from the original library and was therefore evaluated separately using its canonical molecular structure. Their predicted pAffinity values and ranking behavior were examined as reference comparisons rather than as experimental validation of the model predictions.

## 5. Conclusions

This study introduced a retrieval-guided transfer-learning framework for low-resource Ebola drug–target affinity prediction in which source-data selection is treated as an explicit component of transfer learning. Under the leakage-controlled, scaffold-based evaluation protocol, targeted source-data selection consistently produced lower mean RMSE than architecture-matched random retrieval across both evaluated source-data budgets and model backbones. The best overall configuration, GraphDTA with protein-similarity-guided retrieval using 50,000 BindingDB interactions, achieved an RMSE of 0.5498±0.1073, compared with 0.6065±0.0642 for the best conventional machine-learning baseline, Extra Trees, and 0.6533±0.0752 for single-stage GraphDTA. These values correspond to reductions in mean RMSE of approximately 9.3% and 15.8%, respectively.

The results further showed that transfer performance depended on the interaction among source-data relevance, source-data quantity, and model architecture. Protein-similarity-guided retrieval produced the lowest mean RMSE for GraphDTA at both source-data budgets, whereas DeepDTA showed architecture-specific differences among the targeted retrieval strategies. Increasing the source-data budget from 50,000 to 300,000 interactions did not uniformly improve predictive performance, indicating that a larger source dataset was not necessarily more beneficial within the evaluated settings. Retrieval characterization and sequence-alignment analyses further confirmed that targeted selection altered the relevance profile of the source data relative to random retrieval.

The downstream compound-prioritization analyses demonstrated how the resulting model can be used to rank candidate compounds across the six Ebola protein targets and to examine approved drugs as computational repurposing candidates. Seed-wise ranking consistency, scaffold characterization, and target-normalized sensitivity analysis provided additional context for interpreting the resulting rankings. These outputs should be regarded as computational prioritizations for subsequent investigation rather than evidence of antiviral efficacy.

Future work should investigate richer retrieval representations, adaptive source-data selection, additional DTA architectures and source-data budgets, independent target-domain datasets, and complementary structural and experimental validation. More broadly, the findings indicate that relevance-guided source selection can improve transfer learning in low-resource drug–target affinity prediction and that the composition of the source data can be as important as its size when transferring from large external bioactivity collections to a specialized target domain.

## Figures and Tables

**Table 1:** Comparison of machine learning, single-stage deep learning, and retrieval-guided transfer-learning methods for Ebola drug–target affinity prediction. The results are reported across five independent random seeds. Lower RMSE and MAE indicate better performance, whereas higher $R^{2}$ and Pearson correlation indicate better predictive performance. Methods within each category are ordered according to mean RMSE. The best result for each metric within each category is shown in bold. ↓ indicates that lower values are better, whereas ↑ indicates that higher values are better.

Model	Retrieval	Source *n*	RMSE ↓	MAE ↓	$R^{2}$↑	Pearson ↑
**Machine Learning**						
Extra Trees	–	–	**0.6065 ± 0.0642**	**0.4406 ± 0.0284**	**0.8570 ± 0.0338**	**0.9272 ± 0.0186**
Random Forest	–	–	0.6125 ± 0.0634	0.4444 ± 0.0304	0.8542 ± 0.0333	0.9261 ± 0.0185
Gradient Boosting	–	–	0.6590 ± 0.0801	0.4715 ± 0.0433	0.8305 ± 0.0459	0.9136 ± 0.0250
KNN	–	–	0.6796 ± 0.0804	0.4870 ± 0.0316	0.8199 ± 0.0466	0.9108 ± 0.0228
SVR	–	–	0.7216 ± 0.0712	0.5607 ± 0.0427	0.7975 ± 0.0472	0.8963 ± 0.0276
ElasticNet	–	–	0.7391 ± 0.0508	0.5704 ± 0.0430	0.7883 ± 0.0362	0.8938 ± 0.0199
Ridge	–	–	1.1806 ± 0.1746	0.8881 ± 0.1101	0.4609 ± 0.1310	0.7853 ± 0.0417
**Single-Stage Deep Learning**						
GraphDTA	–	–	**0.6533 ± 0.0752**	0.4610	0.8315	0.9148
DeepDTA	–	–	0.6537 ± 0.0556	**0.4567**	**0.8342**	**0.9158**
**Retrieval-Guided Transfer Learning**						
GraphDTA	Protein	50k	**0.5498 ± 0.1073**	0.3634 ± 0.0654	**0.8809 ± 0.0452**	**0.9395 ± 0.0237**
DeepDTA	Hybrid	50k	0.5520 ± 0.0917	0.3634 ± 0.0519	0.8797 ± 0.0411	0.9386 ± 0.0220
GraphDTA	Protein	300k	0.5542 ± 0.0872	0.3929 ± 0.0488	0.8802 ± 0.0365	0.9392 ± 0.0200
DeepDTA	Protein	50k	0.5576 ± 0.0816	**0.3449 ± 0.0511**	0.8767 ± 0.0419	0.9369 ± 0.0217
GraphDTA	Compound	300k	0.5769 ± 0.0835	0.3998 ± 0.0576	0.8705 ± 0.0368	0.9340 ± 0.0205
DeepDTA	Compound	300k	0.5774 ± 0.0997	0.3802 ± 0.0620	0.8687 ± 0.0477	0.9328 ± 0.0244
DeepDTA	Hybrid	300k	0.5885 ± 0.1081	0.3926 ± 0.0567	0.8622 ± 0.0560	0.9295 ± 0.0296
GraphDTA	Hybrid	300k	0.5894 ± 0.0624	0.3953 ± 0.0403	0.8644 ± 0.0331	0.9301 ± 0.0175
GraphDTA	Compound	50k	0.5938 ± 0.0786	0.4118 ± 0.0472	0.8618 ± 0.0406	0.9304 ± 0.0210
DeepDTA	Compound	50k	0.5957 ± 0.1057	0.3854 ± 0.0652	0.8582 ± 0.0568	0.9281 ± 0.0286
DeepDTA	Protein	300k	0.6014 ± 0.0989	0.3946 ± 0.0410	0.8566 ± 0.0500	0.9262 ± 0.0262
GraphDTA	Hybrid	50k	0.6062 ± 0.0706	0.4163 ± 0.0400	0.8575 ± 0.0329	0.9281 ± 0.0164
DeepDTA	Random	300k	0.6168 ± 0.0702	0.4297 ± 0.0344	0.8521 ± 0.0341	0.9241 ± 0.0180
GraphDTA	Random	300k	0.6195 ± 0.0954	0.4264 ± 0.0589	0.8496 ± 0.0494	0.9228 ± 0.0272
GraphDTA	Random	50k	0.6356 ± 0.0741	0.4538 ± 0.0497	0.8425 ± 0.0402	0.9187 ± 0.0220
DeepDTA	Random	50k	0.6643 ± 0.0645	0.4594 ± 0.0299	0.8268 ± 0.0464	0.9112 ± 0.0249

**Table 2:** Characteristics of the BindingDB source subsets selected using different retrieval strategies and source-data budgets. Values are reported as mean ± standard deviation across five independent runs.

Source	Retrieval	Overlap with Others	Protein Similarity to Ebola	Compound Similarity to Ebola
50k	Random	0.0453±0.0003	0.0727±0.0003	0.2257±0.0026
	Protein	0.0485±0.0004	0.1793±0.0010	0.2347±0.0026
	Compound	0.0484±0.0007	0.0738±0.0004	0.3026±0.0046
300k	Random	0.3467±0.0004	0.0703±0.0003	0.2254±0.0026
	Protein	0.3418±0.0006	0.0971±0.0008	0.2260±0.0029
	Compound	0.3414±0.0004	0.0705±0.0003	0.2477±0.0031

Note: Bold values indicate the highest similarity to the Ebola target domain for the corresponding similarity metric within each source-data budget.

**Table 3:** Top 20 compound–target predictions from the virtual-screening analysis in Case Study I. Predicted pAffinity and standard deviation (SD) were calculated across five independently trained models. Agreement indicates the number of seed-specific models in which the compound–target pair appeared among the top-ranked predictions for the corresponding target.

Rank	ChEMBL ID	PubChem CID	Target	pAffinity	SD	Scaffold	Agreement
1	CHEMBL3645734	59533400	sGP	9.076	0.300	D	2/5
2	CHEMBL4858949	164616392	sGP	9.070	0.297	D	2/5
3	CHEMBL4858040	132016497	sGP	9.044	0.192	R	3/5
4	CHEMBL3645737	59533285	sGP	9.029	0.321	U	2/5
5	CHEMBL4871101	–	sGP	9.001	0.155	Q	2/5
6	CHEMBL4469438	71571142	sGP	8.986	0.279	E	2/5
7	CHEMBL4874709	121273823	sGP	8.930	0.121	I	1/5
8	CHEMBL4876200	164625740	sGP	8.921	0.201	S	2/5
9	CHEMBL3645665	59533382	sGP	8.884	0.225	U	1/5
10	CHEMBL3645729	59533233	sGP	8.870	0.121	U	0/5
11	CHEMBL3645738	59533209	sGP	8.855	0.290	U	1/5
12	CHEMBL3645664	59533350	sGP	8.854	0.286	S	1/5
13	CHEMBL411406	16134139	sGP	8.823	0.347	T	2/5
14	CHEMBL3645697	59533295	sGP	8.822	0.261	Z	0/5
15	CHEMBL4114169	89404446	sGP	8.821	0.295	A	1/5
16	CHEMBL4878302	164625830	sGP	8.820	0.525	E	3/5
17	CHEMBL3645667	59533229	sGP	8.816	0.229	U	0/5
18	CHEMBL4860271	164618098	sGP	8.782	0.428	E	1/5
19	CHEMBL3645629	59533355	sGP	8.780	0.144	U	0/5
20	CHEMBL3645666	59533346	sGP	8.779	0.264	U	1/5

**Table 4:** Highest-ranked compound–target prediction for each of the six Ebola protein targets evaluated in Case Study I. Dataset records indicate the number of compound–protein interactions associated with each target in the complete target-domain dataset. Agreement denotes the number of independently trained models in which the compound–target pair appeared among the top-ranked predictions for the corresponding target.

Target	ChEMBL ID	pAffinity	SD	Agreement	Dataset Records
L protein (RdRp)	CHEMBL4114169	8.543	0.393	3/5	1724
Nucleoprotein (NP)	CHEMBL4281745	8.376	0.078	4/5	779
Envelope glycoprotein (GP)	CHEMBL4114169	8.245	0.274	2/5	55
GP (truncated)	CHEMBL3645665	8.053	0.274	3/5	45
Secreted glycoprotein (sGP)	CHEMBL3645734	9.076	0.300	2/5	29
VP35 polymerase cofactor	CHEMBL4281745	8.755	0.491	3/5	33
**Six screening targets**					**2665**

**Table 5:** Approved-drug screening and target-normalization sensitivity analysis. (A) Top-10 compound–target predictions ranked by raw ensemble-mean predicted pAffinity. Seed agreement indicates the number of seed-specific rankings in which each compound–target pair appeared among the top predictions. (B) Top-10 compound–target predictions obtained after within-target normalization of the ensemble-mean predictions.

**(A) Raw Affinity Ranking**					
**Rank**	**Drug**	**Target**	**Pred. pAffinity**	**SD**	**Seed Agreement**
1	Grazoprevir anhydrous	sGP	9.478	0.866	5/5
2	Simeprevir	sGP	9.477	0.584	5/5
3	Voxilaprevir	sGP	9.398	0.704	4/5
4	Voxilaprevir	L RdRp	9.118	0.539	5/5
5	Pibrentasvir	sGP	9.021	0.609	3/5
6	Glecaprevir	sGP	9.009	0.950	4/5
7	Grazoprevir anhydrous	VP35	9.005	0.968	5/5
8	Grazoprevir anhydrous	L RdRp	8.943	0.477	5/5
9	Voxilaprevir	GP	8.784	0.492	5/5
10	Glecaprevir	L RdRp	8.783	0.687	4/5
**(B) Target-Normalized Ranking**					
**Rank**	**Drug**	**Target**	**Normalized z**	**Pred. pAffinity**	**Seed Agreement**
1	Grazoprevir anhydrous	NP	9.304	8.305	5/5
2	Voxilaprevir	NP	8.901	8.159	5/5
3	Simeprevir	NP	7.187	7.541	2/5
4	Grazoprevir anhydrous	VP35	6.838	9.005	5/5
5	Glecaprevir	NP	6.832	7.412	3/5
6	Voxilaprevir	L RdRp	6.780	9.118	5/5
7	Velpatasvir	NP	6.546	7.309	2/5
8	Grazoprevir anhydrous	L RdRp	6.467	8.943	5/5
9	Voxilaprevir	GP	6.467	8.784	5/5
10	Grazoprevir anhydrous	GP	6.408	8.756	5/5

**Table 6:** Predicted affinities and raw screening ranks for selected compounds previously investigated in the context of Ebola virus and additional antiviral comparators. For each compound, the target associated with the reported prediction is shown.

Drug	Best Target	pAffinity	SD	Raw Rank
Daclatasvir	Secreted glycoprotein (sGP)	8.179	1.141	27
Remdesivir	Secreted glycoprotein (sGP)	6.682	0.735	605
Amiodarone	Secreted glycoprotein (sGP)	6.175	0.568	2033
Clomiphene	Envelope glycoprotein (GP)	6.100	0.262	2372
Tilorone ^a^	Envelope glycoprotein (GP)	5.903	0.410	1038 ^b^
Toremifene	Envelope glycoprotein (GP)	5.872	0.411	3913
Chloroquine	Envelope glycoprotein (GP)	5.812	0.389	4445
Bepridil	Envelope glycoprotein (GP)	5.712	0.304	5402
Sertraline	Envelope glycoprotein (GP)	5.529	0.354	7802

^a^ Tilorone was evaluated separately because it was not included in the FDA-approved drug library. ^b^ Tilorone rank was calculated separately within the corresponding target distribution.

**Table 7:** ChEMBL provenance of the retrieved Ebola-related quantitative bioactivity records. Activity types correspond to the original ChEMBL records prior to construction of the sequence-level modeling dataset.

Target ID	Target	Type	Activity	Assays	Records
CHEMBL4434621	Ebola virus	Organism	EC_50_/IC_50_	122	757
CHEMBL4513181	Zaire Ebola virus	Organism	EC_50_/IC_50_	17	99
CHEMBL5291667	Sudan Ebola virus	Organism	EC_50_	6	24
CHEMBL6066662	Tai Forest Ebola virus	Organism	EC_50_	2	14
CHEMBL4105829	Envelope glycoprotein	Protein	EC_50_/$K_{d}$	6	11
CHEMBL6195460	Reston Ebola virus	Organism	EC_50_	1	5
**Total**				**154**	**910**

**Table 8:** Protein-level composition of the final Ebola DTA dataset. Less-represented protein sequences are grouped under “Other viral proteins” for concise reporting.

Protein	Pairs	Compounds	Sequences	Median pAffinity
L protein (RdRp)	1724	1341	9	7.469
Nucleoprotein (NP)	779	775	4	4.724
Other viral proteins	177	153	12	4.947
Envelope glycoprotein (GP)	55	55	1	5.676
Envelope GP (truncated)	45	45	1	6.244
VP35 polymerase cofactor	33	33	1	4.220
Secreted glycoprotein (sGP)	29	29	2	8.585
**Total**	**2842**	**2428**	**30**	–

## Data Availability

The BindingDB dataset used as the source domain in this study is publicly available from the BindingDB database (https://www.bindingdb.org, accessed on 11 September 2026). The Ebola drug–target affinity dataset was compiled from publicly available experimentally measured interactions as described in the manuscript. The source code, trained models, and scripts required to reproduce the experiments and virtual-screening analyses are publicly available at https://github.com/m-mofareh/ebola-retrieval-transfer-learning-v2, accessed on 11 September 2026.
